# Prevalence and factors associated with postpartum depression during the COVID-19 pandemic among women in Guangzhou, China: a cross-sectional study

**DOI:** 10.1186/s12888-020-02969-3

**Published:** 2020-11-25

**Authors:** Peiqin Liang, Yiding Wang, Si Shi, Yan Liu, Ribo Xiong

**Affiliations:** 1 Department of gynecology &obstetrics, The Third Affiliated Hospital of Southern Medical University, 183#West Zhongshan Avenue, Guangzhou, Guangdong province China; 2 Department of rehabilitation, Nanhai Hospital, Southern Medical University, 28#Liguan Road, Lishui County, Foshan City, Guangdong Province China

**Keywords:** Coronavirus, Postpartum depression, Epidemic, Risk factors, China

## Abstract

**Background:**

The 2019 coronavirus disease (COVID-19) is a public health emergency of international concern. To date, there are limited studies that have investigated the impact of COVID-19 pandemic on mental health among female population. Therefore, the study aims to investigate the prevalence of postpartum depression (PPD) and it’s related factors among women in Guangzhou, China, during the COVID-19 pandemic.

**Methods:**

A cross-sectional study was performed from 30th March 2020 to 13th April 2020 using anonymous online questionnaire among 864 women at 6–12 weeks postpartum. The Chinese version of Edinburgh Postnatal Depression Scale and a questionnaire regarding associated factors were administered to all participants. Multivariate logistic regression was used to determine factors that were significantly associated with PPD.

**Results:**

The prevalence of PPD among women at 6–12 weeks postpartum was 30.0%. A multivariate logistic regression model identified significant factors as: immigrant women, persistent fever, poor social support, concerns about contracting COVID-19 and certain precautionary measures.

**Conclusions:**

The findings suggest the need for policies and interventions to not only mitigate the psychological impacts but also target disadvantaged sub-groups of women following childbirth during the COVID-19 pandemic.

## Background

The 2019 coronavirus disease (COVID-19) is a highly infectious disease and have posed a global health threat [1]. Since the emergence of COVID-19 infection in Wuhan, China on December 2019, it has rapidly spread across China and other countries around the world [2]. On January 30th 2020, the World Health Organization (WHO) declared the outbreak of the COVID-19 as a public health emergency of international concern [3]. Since the outbreak, the Chinese government has taken a swift move to reduce the spread of the virus. As of 19th March, zero domestic infection was recorded for the first time since it’s outbreak in China [4].

The ongoing COVID-19 pandemic is not only threatening people’s physical health but also inducing fear and helplessness. Previous research has explored such psychological effect during the outbreaks of infection [5–7]. During the Severe Acute Respiratory Syndrome (SARS) outbreak, 17.3% of heath workers had experienced mental symptoms [5]. During one influenza break, around 10 ~ 30% of general population were concerned about the possibility of contracting the disease [6]. Similarly, the impacts of COVID-19 pandemic on mental health including depression and negative assessment have also been recorded [7]. Risk factors such as being female, student status, specific physical symptoms, poor self-rated health status and increased self-blame were associated with a higher risk of COVID-19-related post-traumatic stress symptoms [7]. For women, the transition to motherhood is a challenging period and has been considered a window of increased vulnerability for the development of mental illness [8]. Therefore, it’s essential to understand the potential psychological changes caused by COVID-19 among perinatal women.

During the postpartum period, women are vulnerable to clinical depression characterized by depressed mood, agitation, disappointment and sleep disorders [9]. Prior research has identified a number of biological, psychological, socioeconomic, and cultural factors that were associated with the development of postpartum depression (PPD) [8]. For example, women with limited financial means are more prone to report PPD [10], perhaps due to the increased financial stress to raise an infant. During the COVID-19 pandemic, it’s imperative to understand the complex interplay of these factors in the development of PPD in Chinese context. Affected by COVID-19, people behave in a more reticent and conservative way such as staying at home with family and reducing get-togethers with friends and relatives [11]. It indicated that people were more likely to gain more support from their family members during this period. On the other hand, restricted travel policy and self-isolation regulations may lead to a more passive lifestyle and a subsequent worsened mental health. To date, there’re limited studies that have investigated the impact of COVID-19 pandemic on mental health of women after delivery.

This study aims to investigate the prevalence of PPD among women in Guangzhou, China, and to explore the related factors of PPD during the COVID-19 pandemic.

## Methods

This study was conducted in Guangzhou, the capital city of Guangdong province and one of the largest metroplis in south China. The city of Guangzhou had a population of more than 16 million, 50% of which were internal migrants [12]. Patients were recruited from the obstetrics unit in the Third Affiliated Hospital of Southern Medical University, a 1000-bed tertiary teaching hospital in Guangzhou, China. In 2019, annual delivery in this unit reached about 4000.

A cross-sectional study was carried out from 30th March 2020 to 13th April 2020 in our hospital using anonymous online questionnaire. The sample size was determined on the assumed effect size of 0.30, referring the study of Gaskin, et al. [13] To obtain reasonable estimates at 95% confidence level and 5% margin of error, a total sample size of 367 was needed. We recruited women at 6–12 weeks after childbirth, 1) Chinese nationality; 2) be living in Guangzhou, China over a month during the COVID-19 period. The COVID-19 period refers to the period from January 2020 until April 2020; 3) providing informed consent in the online questionnaire. Women who had a history or family history of psychiatric disorders were excluded. Women who returned incomplete questionnaires were also excluded. A total of 887 candidates were invited to participate; 23 women refused to be enrolled in and 19 women returned incomplete questionnaire, resulting 845 eligible women. This study included 845 women, allowing detection of significant differences with a power of 0.88 calculated by GPower software.

Data were collected using an anonymous structured questionnaire. The Chinese version of Edinburgh Postnatal Depression Scale (EPDS) was employed to assess PPD with a threshold of 10. The sensitivity and specificity of Chinese version have been found to be 0.82 and 0.86 respectively which were comparable to the original scale [14]. Women with a score of 10 or more were then experienced severity evaluation using Hamilton Depression Rating Scale for depression. A score of ≥7, ≥17 and ≥ 24 were used to identify mild, moderate and severe depression respectively [15].

The questionnaire was based on literature [8] and reviewed by experts in obstetrics and epidemiology from our university. The questionnaire was consisted of the following information: 1) socio-demographic characteristics such as age, employment, annual household income, and household registration place; 2) obstetric data and physical symptoms, such as parity, mode of delivery, pregnancy associated diseases, delivery associated diseases, fever, cough, sore throat, as well as persistent fever and cough or difficulty in breathing; 3) social profiles such as perceived family income sufficiency, family socio-economic status, social support and marital relationship. 4) concerns about COVID-19, such as level of confidence in diagnosis, likelihood of self and other family members contracting COVID-19 and likelihood of surviving if infected; 5) precautionary measures against COVID-19, such as avoidance of sharing of utensils (e.g., chopsticks) during meals, covering mouth when coughing and sneezing, and the average number of hours staying at home per day to avoid COVID-19. Social support was measured by Likert scale, an instrument composed of three subscales that can be classified into informational support, emotional support and household activity support. It’s reliability and validity have been confirmed in a previous study [16]. As the Chinese government recommended the public to isolate themselves at home, women were asked to complete an online questionnaire through an online survey platform. The data collection phase was completed with the help of six nurses and 4 post-graduates. They were trained for one afternoon by the principal investigator covering quality control, completeness of information and research ethics. All completed questionnaires were checked for completeness and consistency.

Ethics approval was obtained from the Research Ethics Board of Southern Medical University.

The primary data was entered into Epidata 3.0 before being exported to SPSS16.0. Chi-square test (for qualitative data) or Student’s *t* test (for quantitative data) was used to assess the differences in socio-demographic characteristics, obstetric data and physical symptoms, social profiles and COVID-19-related factors between women who had PPD and those who did not. The independent variables that were significantly associated (*P*<0.05) with PPD were considered as possible contributing factors and entered into a multivariate logistic regression model. The model controlled for household registration in Guangzhou, persistent fever, social support, likelihood of contracting COVID-19 during the current outbreak, and avoiding sharing of utensils. Odds Ratios (ORs) with 95% confidence intervals (95%CIs) were calculated to measure the strength of association. A *P* value <0.05 was considered significant in the analysis.

## Results

Of the 845 women who were enrolled in the assessment, we have reported that 253 women were screened positive for PPD with EPDS at a cut-off point of 10, thus resulting in a prevalence of 30.0%. The number of women who had mild, moderate and severe PPD was 125 (14.8%), 91(10.8%), 37(4.4%) respectively.

Of all the respondents, 42.0% were aged between 25 and 29 years. More than three-quarters (79.7%) of the participants had senior high school or college level education. 73.1% of the participants were unmarried. 47.2% of participants reported annual household income of 15,000–19,999 dollars. Distribution by place of household registration showed that 52.1% of the participants were immigrants.

The association between socio-demographic characteristics and PPD was presented in Table 1. Immigrant women were significantly more likely to report PPD compared with their local counterparts.

**Table 1:** Socio-demographic features of women with PPD

Factors	EPDS < 10 (592)		EPDS ≥ 10 (253)		***χ***^***2***^	***P***
	n	%	n	%		
Age					0.153	0.985
≤ 24	95	16.0	41	16.2		
25 ~ 29	248	41.9	107	42.3		
30 ~ 34	161	27.2	70	27.7		
≥ 35	88	14.9	35	13.8		
Educational level					0.054	0.973
Junior high school or less	121	20.4	50	19.8		
Senior high school	203	34.3	88	34.8		
College or more	268	45.3	115	45.4		
Employment					0.264	0.612
Yes	436	73.6	182	71.9		
No	156	26.4	71	28.1		
Annual household income					0.240	0.971
$10,000 ~ 14,999	141	23.8	58	22.9		
$15,000 ~ 19,999	278	47.0	121	47.8		
$ 20,000 ~ 24,999	122	20.6	54	21.3		
≥ $25,000	51	8.6	20	7.9		
Household registration in Guangzhou					4.597	0.035
Yes	298	50.3	107	42.3		
No	294	49.7	146	57.7		

Analysis of obstetric data, physical symptoms within the past 14 days and probable PPD was shown in Table 2. Women who reported persistent fever were significantly more likely to develop PPD.

**Table 2:** Obstetric data and physical symptoms of women with PPD

Factors	EPDS < 10 (592)		EPDS ≥ 10 (253)		***χ***^***2***^	***P***
	n	%	n	%		
Parity					0.000	1.000
1	278	46.9	119	47.0		
≥ 2	314	53.1	134	53.0		
Mode of delivery					0.070	0.817
Vaginal	364	61.5	158	62.5		
Caesarean	228	38.4	95	37.5		
Pregnancy related disease					0.212	0.632
Yes	109	18.4	50	19.8		
No	483	81.6	203	80.2		
Delivery related disease					0.351	0.607
Yes	91	15.4	43	17.0		
No	501	84.6	210	83.0		
Persistent fever(>37.4 °C for at least 1 day)					709.1	0.000
Yes	18	3.0	241	95.3		
No	574	97.0	12	4.7		
Cough					0.025	0.867
Yes	163	27.5	71	28.1		
No	429	72.5	182	71.9		
Sore throat					0.072	0.806
Yes	408	68.9	172	68.0		
No	184	31.1	81	32.0		
Persistent fever and cough or difficulty in breathing					1.388	0.235
Yes	36	6.1	21	8.3		
No	556	93.9	232	91.7		

For social profiles, Table 3 showed lower social support was significantly associated with the development of PPD.

**Table 3:** Social profiles of women with PPD

Factors	EPDS < 10 (592)		EPDS ≥ 10 (253)		***χ***^***2***^***/t***	***P***
	n	%	n	%		
Perceived family income sufficiency					0.066	0.967
Not enough	232	39.2	100	39.5		
Just enough	318	53.7	134	53.0		
More than enough	42	7.1	19	7.5		
Family socio-economic status					0.187	0.911
Low	108	18.2	45	17.8		
Middle	264	44.6	110	43.5		
High	220	37.2	98	38.7		
Social support	41.03 ± 6.78		38.70 ± 9.16		−18.004	0.000
Marital relationship					0.271	0.873
Poor	89	15.0	36	14.2		
Moderate	367	62.0	155	61.3		
Satisfying	136	23.0	62	24.5		

Table 4 represented the relationship between concerns about COVID-19 and PPD. Variables were not associated with PPD, with the exception of concerning on contracting COVID-19 during the current outbreak which was significantly associated with PPD.

**Table 4:** Association between concerns about COVID-19 and PPD

Factors	EPDS < 10 (592)		EPDS ≥ 10 (253)		***χ***^***2***^	***P***
	n	%	n	%		
Level of confidence in doctor’s ability to diagnose or recognize					1.129	0.569
High	314	53.1	136	53.8		
Middle	228	38.5	101	39.9		
Low	50	8.4	16	6.3		
Likelihood of contracting COVID-19 during the current outbreak					104.1	0.000
High	105	17.7	112	44.3		
Middle	174	29.4	96	37.9		
Low	313	52.9	45	17.8		
Concerns about other family members contracting COVID-19					0.103	0.960
High	211	35.6	90	35.6		
Middle	200	33.8	88	34.8		
Low	181	30.6	75	29.6		
Likelihood of surviving if infected with COVID-19					0.438	0.803
High	479	80.9	206	81.4		
Middle	94	15.9	41	16.2		
Low	19	3.2	6	2.4		

Regarding the precautionary measures adopted by the respondents, avoiding the sharing of utensils (e.g, chopsticks) during meals was significantly associated with the lower rates of PPD (Table 5).

**Table 5:** Association between precautionary measures and PPD

Factors	EPDS < 10 (592)		EPDS ≥ 10 (253)		***χ***^***2***^	***P***
	n	%	n	%		
Covering mouth when coughing or sneezing					0.501	0.778
Always	409	69.1	178	70.4		
Sometime	140	23.6	60	23.7		
Occasionally	43	7.3	15	5.9		
Washing hands with soap					0.255	0.881
Always	396	66.9	170	67.2		
Sometime	168	28.4	73	28.8		
Occasionally	28	4.7	10	4.0		
Wearing a mask regardless of the presence or absence of symptoms					0.089	0.967
Always	486	82.1	209	82.6		
Sometime	95	16.0	40	15.8		
Occasionally	11	1.9	4	1.6		
Avoiding sharing of utensils (e.g, chopsticks) during meals					145.3	0.000
Always	318	53.7	41	16.2		
Sometime	173	29.2	75	29.6		
Occasionally	101	17.1	137	54.2		
Average number of hours staying at home per day to avoid COVID-19					0.399	0.819
20 ~ 24 h	189	31.9	82	32.4		
10 ~ 19 h	293	49.5	120	47.4		
0 ~ 9 h	110	18.6	51	20.2		

Table 6 showed the factors associated with PPD during COVID-19 pandemic in the study site. Immigrant women were 3.1 times significantly more likely to develop PPD compared to local women (OR = 3.135, 95% CI: 2.759 ~ 3.428). In relation to physical symptoms, women who reported persistent fever were 2.1 times significantly more likely to develop PPD compared to women who had no fever (OR = 2.084, 95% CI: 1.737 ~ 2.539). Similarly, women who had lower social support were at greater risk of PPD (OR = 3.478, 95% CI: 2.259 ~ 3.701). In relation to concerns about COVID-19, women who perceived higher likelihood of contracting COVID-19 during the current outbreak were 3.3 times significantly more likely to develop PPD compared to women who perceived their likelihood of contraction was low (OR = 3.276, 95% CI: 2.611 ~ 3.589). The likelihood of PPD was also varied by their dining customs. Women who preferred to using serving utensils (e.g, chopsticks) during meals were 37% significantly less likely to develop PPD compared to those who used sharing utensils (OR = 0.672, 95% CI: 0.251 ~ 0.907).

**Table 6:** Multivariate logistic regression analysis of impact factor of PPD

Covariates	***B***	***S.E***	***Wald***	***P***	***OR***	***95%CI***
Household registration place in Guangzhou						
Yes	–	–	21.049	–	–	–
No	0.067	0.236	28.730	0.000	3.135	2.759 ~ 3.428
Persistent fever (>37.4 °C for at least 1 day)						
Yes	0.018	0.225	11.341	0.000	2.084	1.737 ~ 2.539
No	–	–	8.709	–	–	–
Social support	0.615	0.349	35.012	0.000	3.478	2.259 ~ 3.701
Likelihood of contracting COVID-19 during the current outbreak						
High	0.541	0.133	21.085	0.000	3.276	2.611 ~ 3.589
Middle	0.677	0.435	18.018	0.721	1.549	0.608 ~ 2.133
Low	–	–	26.547	–	–	–
Avoiding sharing of utensils (e.g, chopsticks) during meals						
Always	0.097	0.169	15.044	0.013	0.627	0.251 ~ 0.907
Sometime	0.008	0.136	29.820	0.156	1.349	0.886 ~ 1.835
Occasionally	–	–	31.472	–	–	–

## Discussion

To our knowledge, this study was among one of the first studies with respect to the psychological responses of the delivery women in mainland China. The prevalence rate of PPD was as high as 30.0%. The present figure was higher than the prevalence of PPD reported in other studies carried out in China using the EPDS scale [17–19]. For example, in Hebei province of north China, 20.3% of women had elevated levels of postpartum depressive symptoms [17]. In Shanghai of east China, the estimated prevalence of PPD 6 weeks after delivery was 11.8% [18]. In Guangzhou of south China, the rate of PPD was 27.4% [19]. However, our result is consistent with reports in Asian countries which indicated the prevalence of PPD ranged from 3.5 to 63.3% [20]. Another cross-sectional study in Brazil also found a similar prevalence of PPD (27.9%) among low income women [10]. Higher rates of PPD in this study may be explained by two evident differences between other studies and the current one. First, the present data were obtained during the COVID-19 pandemic. Although the COVID-19 outbreak in Guangzhou may not be regarded as severe, the number of imported cases is increasing during the time the study was conducted. According to Behavioral Immune System theory, people are likely to develop negative emotions and avoidant behaviors when faced with public health emergencies [11]. The uncertainty and unpredictability of COVID-19 may cause cognitive dissonance and insecurity, thus providing a feeling of mental discomfort. In addition, with the closure of schools and business as well as social-distancing regulations, negative emotions experienced by individuals are compounded. Therefore, these COVID-19 related factors have helped to add the stressful impact on women’s mental health. Second, the present study was entirely drawn from Guangzhou, one of the most affluent metropolis in south China. As Guangzhou is one of the major air transportation hubs with more than 130 international flights connecting main countries in the world, the potential impact of global COVID-19 outbreak is high. Moreover, with the lockdown eased gradually to ensure smooth resumption of work and production, internal migrants who were originally from the large poor and rural areas in the western and central inland provinces migrated to the southern developed regions, such as Guangzhou for better job opportunities and income. The convenience of long-distance travel could increase the incidence of local cases through respiratory droplets (e.g., from exhalation sneeze) and contact routes [21]. And the possibility of transmission by asymptomatic carriers could further enhance it’s spread [21]. Such occurrences of both imported cases and domestically transmitted cases have significant potential for psychological contagion, resulting in widespread fear, helplessness, and a variety of adverse mental health outcomes [7].

The findings of this study showed that immigrant women were significantly more likely to develop PPD compared to local registered women, which was consistent with the findings of previous studies that immigrant women were at increased risk of depression perinatally [22]. Due to long-standing household registration policy in China, internal migrants do not have the same rights and benefits as local residents in a variety of areas, such as healthcare, social services, education and employment. We hypothesized social exclusion resulting from this policy as well as other economic and cultural factors have an impact on mental health of immigrants. We also reported a strong association between social support and PPD which was consistent with a previous study [19]. One possible reason is that during the pandemic, the pace of the whole society is slowed down. This could have been created more opportunities and time for family member to support and care for each other. In addition, communication with community members and friends was increased because people were asked to stay at home instead of going to public places. These positive impacts may have helped women cope with the challenges surrounding the postpartum period.

In addition, we explored the relationship between mental health and physical symptoms as well as concerns about COVID-19 among delivery women during the pandemic. The presence of a persistent fever was significantly associated with PPD. Similarly, a higher perceived likelihood of contracting COVID-19 during the current outbreak was significantly associated with PPD. Amid this moment, women were bombarded with various discomforting network information about COVID-19, including clinical signs, routes of transmission, medicines or vaccines, et al. After presentation to the clinic with a fever, they may be sent home, hospitalized for further observation, or quarantined. Some evidences suggest that up-to-date and accurate information during the pandemic is responsible for lower levels of stress, anxiety and depression [7]. Moreover, higher satisfaction with the health information received by the whole population is contributing to the reduced impact of rumors and this may avoid adverse psychological reactions.

Also, our findings suggest that precautionary measures adopted to prevent the spread of COVID-19 have had a positive psychological effect. Women who had avoided the sharing of utensils (e.g, chopsticks) during meals were significantly less likely to develop PPD. There has been no evidence to suggest the reason for the difference, but saliva is one of the most common ways for food-borne diseases to spread. Communal eating habits have been a part of Chinese culture for centuries. Chinese people prefer to using chopsticks to pick up food commonly shared in the table during meal times to show their respect. The experiences of the SARS-COV epidemic in 2013 may have changed the perception of the general public towards precautionary measures. Many cities in China have already launched initiatives to order separate meals. As this healthy habit is related to people’s health and safety during the pandemic, it’s not unexpected that avoidance of sharing utensils during meals is significantly associated with less psychological impact on women.

Our findings will provide vital guidance for health care professionals to tackle mental health issues among delivery women during a pandemic. First, health authorities need to identify high-risk groups such as immigrants for early intervention. Second, accurate and up-to-date health information during the pandemic needs to be provided in order to alleviate the concern and reduce the impact of rumors. Third, government and health authorities need to expand public awareness of healthy lifestyle.

This study has several limitations. First, a cross-sectional study did not allow for establishing causal relationships between PPD and the factors associated with it. Second, a self-reported scale was employed to define PPD instead of clinician administered structured interview. Participants might have provided responses they feel socially desirable. Third, the short time frame might not allow us to observe it’s long-term impacts on mental health among delivery women.

## Conclusion

During the COVID-19 pandemic in China, about one-third of the women reported PPD. Immigrants, persistent fever, lower social support and higher level of concerning about contracting COVID-19 were associated with higher risk of PPD. Certain precautionary measures were associated with a lower risk of PPD. Since the COVID-19 is going on, our findings can be used to formulate psychological interventions to minimize depression and improve psychological resilience among women following childbirth.

## Data Availability

The datasets used and/or analyzed during the current study are available from the corresponding author on reasonable request.

## Abbreviations

COVID-19: 2019 Coronavirus Disease
PPD: Postpartum depression
EPDS: Edinburgh Postnatal Depression Scale
WHO: World Health Organization
SARS: Severe Acute Respiratory Syndrome
